# Enzyme inhibitory activity of marine alkaloid aaptamines for neurodegenerative diseases: an *in silico* study

**DOI:** 10.1098/rsos.250697

**Published:** 2025-07-16

**Authors:** Thi Le Anh Nguyen, Hoang Linh Nguyen, Thi My Pham, Dinh Hieu Truong, Thi Chinh Ngo, Mai Suan Li, Duy Quang Dao

**Affiliations:** ^1^ Institute of Research and Development, Duy Tan University, Danang, Vietnam; ^2^ School of Engineering and Technology, Duy Tan University, Danang, Vietnam; ^3^ Institute of Fundamental and Applied Sciences, Duy Tan University, Ho Chi Minh City, Vietnam; ^4^ Faculty of Pharmacy, School of Medicine and Pharmacy, Duy Tan University, Danang, Vietnam; ^5^ Institute of Physics, Polish Academy of Sciences, Warsaw, Poland

**Keywords:** aaptamines, enzyme inhibition, docking, molecular dynamics, ADMET, neurodegenerative diseases

## Abstract

The enzyme inhibitory activities of a dataset of 28 aaptamines are performed to identify potential multifunctional drugs for neurodegenerative diseases (NDs). First, the drug-like properties and pharmacokinetic (ADMET) study excluded seven molecules, mostly for the non-permeability of the blood–brain barrier. The binding activities of the remaining 21 candidates towards acetylcholinesterase (AChE), monoamine oxidase B (MAOB) and catechol-O-methyltransferase (COMT) enzymes are initially screened by molecular docking. The top binding complexes (**A12**@MAOB, **A24**@COMT and **A27**@AChE) are simultaneously studied by molecular dynamics in water for 500 ns time-scale and compared with the references such as safinamide (SAG), tolcapone (TOL) or donepezil (DON). The results show that two aaptamines **A12** and **A27** are well-positioned within the active pocket of the enzymes, exhibiting structural stability, with a root mean square deviation of about 0.15–0.2 nm. MM-PBSA calculation indicates that the binding energy of the ligands to the corresponding targets is equal to (**A12** versus SAG) or much lower than the references (**A24** versus TOL and **A27** versus DON). The van der Waals interactions contribute more strongly to enzyme binding than the electrostatic energy. The study results suggest that **A27** (lowest binding energy, −170.42 ± 14.24 kJ mol^−1^) is the most prominent aaptamine candidate for the treatment of NDs.

## 1. Introduction

Neurodegenerative diseases (NDs) are a group of disorders characterized by the progressive degeneration of the structure and function of the nervous system via the degeneration and loss of specific neuronal cells, the building blocks of the brain and spinal cords. As neurons lose their functions and die, patients experience a gradual decline in motor control, cognitive function and memory [1]. The two most common forms of NDs are Alzheimer’s disease (AD) and Parkinson’s disease (PD) [2], which are about 60−70 and 15% of ND cases, respectively. While AD typically begins with mild memory loss and confusion, it gradually worsens over time, leading to severe cognitive and functional impairments. In contrast, PD primarily affects movement, with typical symptoms such as dyskinesia, muscular rigidity, resting tremors and postural instability [3]. Particularly, AD is the most common cause of dementia, cognitive decline and the loss of the ability to carry out daily activities. According to the 2015 report by Alzheimer’s Disease International, AD-induced dementia affected about 46 million people worldwide at that time, with projections indicating an increase to 131 million by 2050. Consequently, NDs pose significant challenges to healthcare systems and socio-economic structures, particularly in developing countries, where much of the burden of care falls on families and caregivers [4].

While the exact causes of AD and PD remain unknown, protein-related pathologies are widely accepted as central to their progression. These include the aggregation of amyloid-beta (Aβ) into oligomers, fibrils and plaques, as well as the aggregation of hyperphosphorylated tau protein into helical filaments, which subsequently form intracellular neurofibrillary tangles [3,5]. The biochemical events underlying AD are believed to be the deficiency and loss of cholinergic synapses, which has led to the development of pharmacological treatments primarily based on acetylcholinesterase (AChE) inhibitors. The four FDA-approved drugs for AD include three AChE inhibitors (donepezil, rivastigmine and galantamine), and memantine, a non-competitive *N*-methyl-ᴅ-aspartame (NMDA) glutamate receptor antagonist. The most common current treatment for AD involves a combination of an AChE inhibitor (donepezil) and memantine, simultaneously targeting the cholinergic and glutamatergic systems [6]. However, given the complex and multifactorial nature of AD pathophysiology, none of the current treatments are truly effective at halting the disease; they only slow its progression. Additionally, AChE inhibitor treatment is associated with several side effects, including nausea, vomiting, diarrhoea, abdominal pain, anorexia, headache or insomnia [7,8]. In PD, typical symptoms relate to the accumulation of α-synuclein protein and the loss of dopaminergic synapse. Because both catechol-O-methyltransferase (COMT) and monoamine oxidase B (MAOB) enzymes are involved in the breakdown and the metabolism of dopamine, the PD treatment can use alternative targets [9–11]. The main treatment for PD currently involves levodopa (L-DOPA), a precursor of dopamine, combined with a COMT inhibitor (tolcapone, entacapone or opicapone), or a MAOB inhibitor (rasagiline, selegiline, safinamide or zonisamide). Although inhibition of COMT and MAOB enzymes has shown effectiveness in treating PD, significant side effects and limitations are reported, including fluctuation in dopamine levels leading to ON-OFF periods, non-motor symptoms or bradykinesia induced by L-DOPA, and hepatoxicity in the case of entacapone.

Importantly, research has long suggested that oxidative stress plays a significant role in the development and progression of NDs [11,12]. Resulting from an imbalance between free radicals and the body’s ability to neutralize them with antioxidants, oxidative stress reflects the unregulated production of reactive free radicals (e.g. reactive oxygen species and reactive nitrogen species) in the body. Its consequence is the pro-oxidation of lipids [12–14], proteins and DNA [15], damaging brain cells, inducing cell death and promoting protein aggregations—hallmark of NDs [16,17]. Chemically, high levels of 4-hydroxy-2,3-nonenal (HNE), resulting from lipid oxidation, hydroxylation, carbonylation and nitration of DNA bases, as well as elevated levels of redox-active metals (e.g*.* Fe, Cu), are observed in AD brains. Similarly, increased levels of malondialdehyde and DNA oxidation products, such as 8-hydroxyadenine or 8-hydroxyguanine, are found in PD brains.

In clinical practice, the implementation of levodopa therapy with COMT and MAOB inhibitors has been shown to reduce free radicals triggered by homocysteine, thereby decreasing oxidative stress via dopamine metabolism [10]. For several decades, intensive efforts have been made to develop AD/PD drugs that combine enzyme inhibition and antioxidant activities, particularly through a multifunctional, multi-target-directed ligand strategy.

From our viewpoint, searching for a better therapeutic solution for NDs from natural sources, such as plants, microorganisms or marine organisms, holds important potential for advancing the field. Indeed, Moodie *et al.* have reported 185 natural cholinesterase inhibitors derived from marine organisms, some of which display inhibitory activity comparable to or exceeding that of currently used clinical inhibitors [18]. In China, sodium oligomannate (GV-971), a mixture of oligosaccharides isolated from the marine algae *Ecklonia kurome*, has been approved for AD treatment since 2019. However, it is only available in China, as the clinical trials have been deemed insufficient for approval in other countries, and the drug’s mechanism of action remains unclear [19]. Recently, Miao *et al.* reported that aaptamine, a compound extracted from marine sponges, shows *in vivo* therapeutic effects in a zebrafish model. In that study, aaptamine acted as a dual AChE and BuChE inhibitor, with treatments of 10 and 20 µM of aaptamines yielding therapeutic effects similar to those of 8 µM donepezil. The dyskinesia recovery rate at the optimal dose reached up to 60%, showing potential use for AD treatment [20]. Additionally, interesting biological activities of the aaptamine derivatives, including antibacterial, anticancer [21,22] and antioxidant activities [23], can bring added values.

In recent years, computer-aided drug design (CADD) [24–26] has significantly advanced the development of pharmaceutical molecules. CADD, including advanced technologies like machine learning and artificial intelligence [27], has demonstrated its value across various stages of drug development, from target identification and hit selection to lead optimization and preclinical evaluation. By 2019, over 70 commercial drugs had been developed with the integration of CADD [28]. Notable successes include HIV-1 protease inhibitors such as raltegravir [29], approved in 2007 and dolutegravir [30] in 2013 for first- and second-line HIV treatments [31]. Another example is the development of tyrosine kinase inhibitors for cancer therapy, ranging from imatinib [32], approved in 1990 for chronic myeloid leukaemia, to zanubrutinib [33], approved in 2023 for mantle cell lymphoma and other B-cell malignancies. Additionally, CADD has played a critical role in the development of anti-influenza drugs like tamiflu, and molnupiravir emergently approved during the COVID-19 pandemic [34]. Particularly, for AD, CADD has been used in advancing β-secretase inhibitors like donanemab and solanezumab into clinical trials [35,36]. Beyond selecting drugs from extensive compound libraries, CADD also facilitates the design of new molecules using AI techniques like fragment-based design [37,38], forecasts the protein structure with high accuracy [39] and repurposes known drugs [40]. With rapid evolution in technology and computing power, CADD has significantly reduced the time and cost of the screening process, paving the way for more efficient small-molecule discovery and approval.

**Figure 1.**
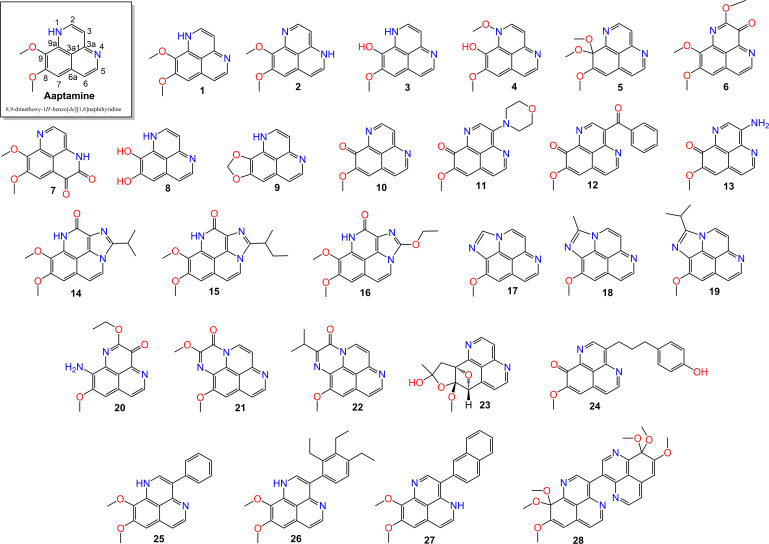
Structures of aaptamines **A1**–**A28** used in this work. Aaptamine **A1** is presented with position numbering.

In this study, we aim to identify compounds with enzyme inhibition activities suitable for ND treatment from natural products by an *in silico* method. Hit compounds are selected from a dataset of 28 aaptamine derivatives (set A, **A1**–**A28**) (figure 1), all of which are natural alkaloids isolated from *Aaptos aaptos* marine sponges collected across the Pacific Ocean (Japan, Taiwan, Indonesia, Malaysia, Vietnam, China) [41–53] and known for their bioactive properties. For this purpose, our study follows a three-tier approach. First, we screen the molecules for physicochemical properties, drug-likeness and pharmacokinetics to select first-range candidates appropriate for drug development. Next, enzyme inhibition activities are assessed through molecular docking to further narrow the candidate list. Finally, molecular dynamics (MD) simulations are performed for more details on conformational changes, stability and interactions that occur in the ligand–enzyme binding of the complexes over a real time scale. The MD results are intentionally compared with the reference systems, via an actual therapeutic method.

## 2. Computational methods

For *in silico* assessment of aaptamines’ drug-like properties, the molecules were first evaluated based on the rule of five (Lipinski’s Rule), followed by an analysis of pharmacokinetics properties (ADMET: absorption, distribution, metabolism, excretion and toxicity). Absorption, distribution and metabolism were predicted using the free Web tool SwissADME (http://www.swissadme.ch/), while excretion and toxicity data were obtained from the pkCSM tool (https://biosig.lab.uq.edu.au/pkcsm/) [54].

Optimized structures of aaptamines in water were obtained using Gaussian G16 Rev. A03 [55]. Density functional theory (DFT) using the M06-2X method [56], combined with the 6-311++G(d,p) basis set, was applied. Solvent effects were studied via an implicit approach with a continuum solvent model SMD [57] for water. Docking studies were performed with Autodock4.2.6 [58]. Three target proteins, including AChE, COMT and MAOB, associated with neural functional activities were investigated. The three-dimensional (3D) structures of these enzymes were obtained from the RCSB database (https://www.rcsb.org/) with PDB IDs 4EY7, 4PYL and 2V5Z, respectively. Before docking, the topology pdbqt files of receptors and ligands were generated via AutoDockTools. The structures of the target enzymes were prepared by removing water and small molecules, followed by the addition of polar hydrogens and Kollman charges. The active pocket was determined by a box with dimensions of approximately 60 × 60 × 60 grid points around the centre grid box. Redocking of the ligands from the crystallized enzyme structures was performed to validate the method. Docking parameters included Lamarckarian genetic algorithm (LGA) with 30 GA runs, a population size of 300, and a maximum number of 10 000 000 evaluations. Ligand–enzyme interactions were analyzed alongside docking scores (kJ mol^−1^) to determine the strongest interacting conformation.

The best docking score complexes were then chosen for MD studies to confirm the aaptamines’ interaction with the target enzymes over time. MD simulations were performed using GROMACS 2023 packages [59], with the AMBER f99SB-ILDN force field [60] and TIP3P water model [61]. Ligands were prepared with Antechamber [62,63] and ACPYPE [64], with the GAFF2 force field and AM1-BCC charge method. AMBER ff99SB-ILDN is a validated choice for ligand binding free energy calculations [65]. The complex was placed in a cubic box at a distance of 1.0 nm from the solute to the box surface and neutralized with Na^+^ and Cl^−^ ions, at a concentration of 0.15 M. Simulations were run at 300 K and 1 atm, with temperature and pressure maintained via V-rescale and Parrinello–Rahman [66] algorithms. System equilibration was carried out in two phases: NVT (250 000 steps, 2 fs per step) and NPT (2 500 000 steps, 2 fs per step). The Verlet cutoff scheme was used for short-range van der Waals interactions (1.2 nm), and the particle mesh Ewald for long-range electrostatic (1.2 nm). Finally, MD simulations were conducted using a leap-frog integrator with a time step of 2 fs over a duration of 500 ns for each trajectory. For each system, at least three independent trajectories were collected. Analyses included root mean square deviation (RMSD), root mean square fluctuation (RMSF), radius of gyration (R_g_), hydrogen-bond, as well as non-bonding interactions. The molecular mechanics Poisson−Boltzmann surface area (MM-PBSA) method was calculated to estimate the binding free energy as follows:


$$\Delta G_{bind}=\Delta E_{elec}+\Delta E_{vdW}+\Delta G_{PB}+\Delta G_{SA}-T\Delta S,$$

where Δ*E*_elec_ and Δ*E*_vdW_ are electrostatic and van der Waals energies, averaged over MD snapshots extracted from the 350−500 ns trajectory window. Δ*G*_PB_ and Δ*G*_SA_ are polar and non-polar solvation energies, respectively. The polar solvation energies were calculated using the Delphi program [67]. The non-polar solvation energies were derived from the solvent accessible surface area (SASA) in Å^2^ using the equation Δ*G*_SA_= γ × SASA with γ = 0.030125 kJ mol^−1^ Å^−2^. The entropic contributions −*T*Δ*S* were obtained via the interaction entropy method [68]. Particularly, each complex system was compared to the corresponding reference. Clustering analysis was performed on MD snapshots extracted from the 350 to 500 ns trajectory interval using the GROMOS algorithm implemented in gmx cluster. RMSD calculations were restricted to Cα atoms of the protein and heavy atoms of the ligand. Visualization of the binding poses of the inhibitors to their targets was illustrated by ChimeraX [69].

## 3. Results and discussion

### 3.1. Drug-likeness and pharmacokinetics

Preliminary information, such as the physicochemical and drug-like properties of the studied compounds, can be quickly achieved beforehand. The physicochemical parameters for compounds of set A (**A1–A28**) are given in electronic supplementary material, table S1. Despite the structural diversity among the aaptamines, we found that the molecular properties of all molecules (except **A28**) comply with Lipinski’s rule of five, suggesting that the studied compounds are bioavailable.

**Figure 2.**
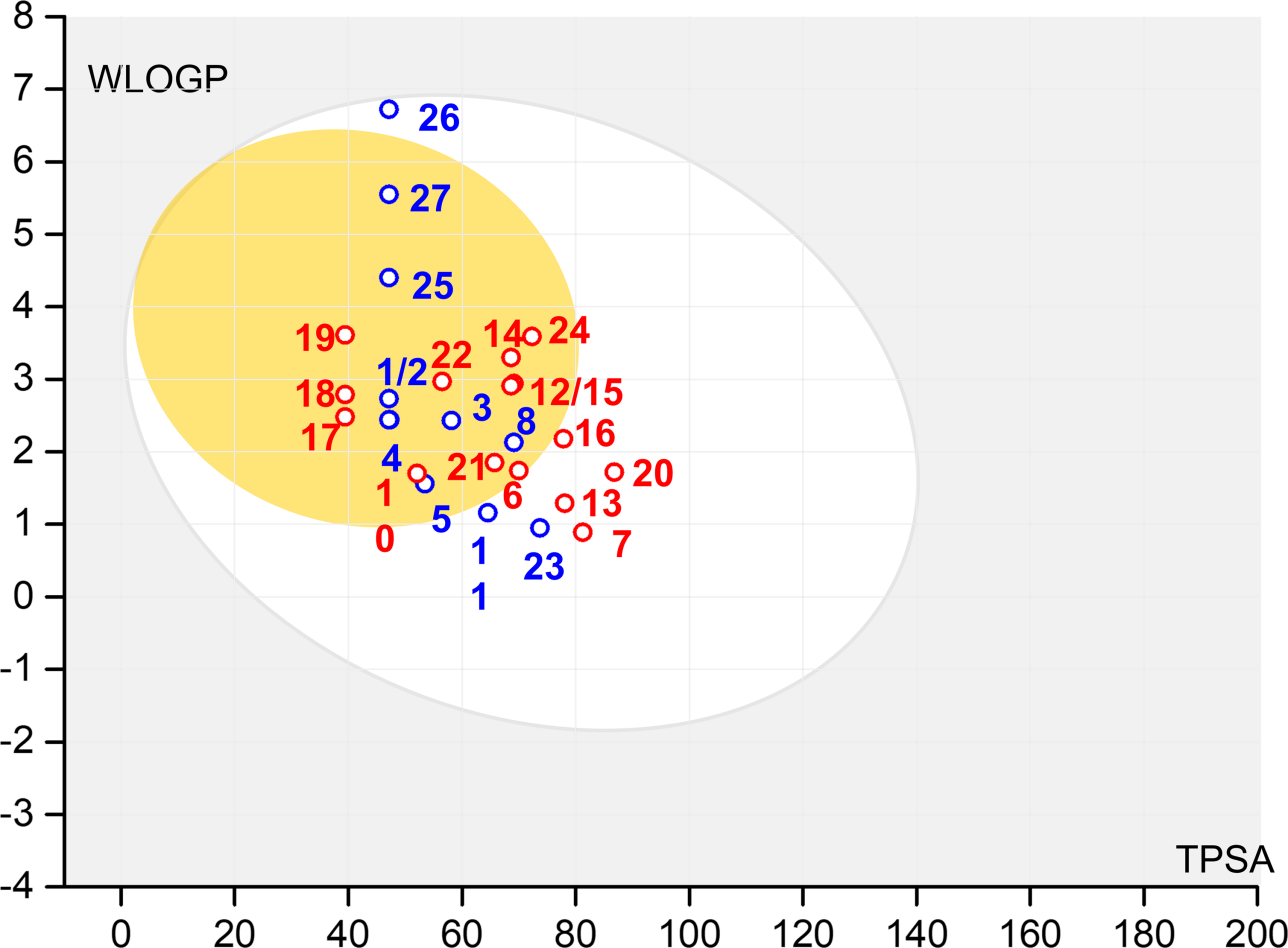
BOILED-Egg prediction of set B (**A1**–**A27**).

Pharmacokinetics predictions (ADMET) for compounds **A1**–**A27** (set B) are given in electronic supplementary material, table S2. All selected molecules exhibit high gastrointestinal absorption. As we aim to identify potential candidates for the treatment of NDs, the blood–brain barrier (BBB) permeability was considered a primary criterion. The selection of potential compounds was supported by the Brain Or IntestinaL EstimateD permeation (BOILED-Egg) model [70], which is based on two key parameters, lipophilicity/polarity (logP) and topological polar surface area (TPSA). According to this model, compounds in the yellow zone have the highest probability of permeating to the brain and gastrointestinal absorption, while those in the white zone are absorbed by the gastrointestinal tract but do not permeate the brain. The blue and red points represent compounds that are substrates, or non-substrates to P-glycoprotein (P-gp), respectively. Figure 2 shows that six molecules (**A7**, **A13**, **A16**, **A20**, **A23** and **A26**) fall within the white region, suggesting they cannot cross into the brain. Therefore, these molecules were excluded from further studies. The 21 other remaining compounds (set C) are located in the yellow region, indicating high probabilities of both brain permeation and gastrointestinal absorption. It is noteworthy that the yolk and white areas are not mutually exclusive.

The metabolism is characterized by Cytochrome P450 (CYP) enzymes. Six key CYP enzymes (CYP1A2, CYP2C9, CYP2C19, CYP2D6, CYP3A4 and CYP3A5) are responsible for the metabolism of over 90% of medications. Among them, CYP2D6 and CYP3A4 are particularly significant due to their role in metabolizing a wide variety of drugs, with studies showing that drug responses can vary among patients of different ethnic backgrounds [71]. Our results indicate that each of the 21 selected aaptamines (set C) inhibits at least two of six key CYP P450 enzymes. Particularly, all 21 aaptamine candidates showed inhibitory activity towards CYP1A2 and CYP3A4.

Post-metabolism excretion/elimination of these compounds was assessed by examining clearance (Cl) and the renal Organic Cation Transporter 2 (OCT-2) substrate index. Total clearance, combining hepatic clearance and renal clearance, was positive for all 21 aaptamines of set C, ranging from 0.091 to 1.088 (log ml/min/kg). All compounds are also classified as a renal OCT-2 substrate. The highest clearance values were observed for **A14**–**A19**, suggesting strong excretion potential.

Regarding the toxicity, multiple criteria were applied, including (i) AMES toxicity, (ii) maximum tolerated dose, (iii) hERG I/II inhibition, (iv) hepatotoxicity, (v) oral rat acute (LD50), (vi) chronic toxicity, (vii) skin sensitization, (viii) *Tetrahymena pyriformis* (*T. pyriformis*) toxicity and (ix) minnow toxicity. The results reveal that most aaptamines are potential mutagenic (AMES positive), with more than half of the compounds being hepatotoxic. However, none of the compounds inhibit hERG I, and only a few (**A14**, **A15**, **A17**, **A19**, **A21**, **A24**–**A27**) inhibit hERG II. In addition, all aaptamines are non-sensitizing to the skin, and most are non-toxic to minnow mice.

In summary, pharmacokinetics predictions suggest that 21 out of the 28 compounds are permeable to the brain and are then selected for further study. It should be noted that all of them can be metabolized by Cytochrome P450 enzymes, and they can also be excreted with positive total clearance values, with some classified as OCT-2 substrates. Toxicity evaluation further shows that the compounds exhibit AMES toxicity, hepatotoxicity, varying levels of hERG I/II inhibition and rat toxicity.

### 3.2. Static interaction and binding energy

Docking studies predict the formation of a stable conformation of enzyme–ligand complexes and help to classify the ligand in terms of binding affinity in static conditions, which is derived from scoring functions. For a docked complex, highly negative binding free energy and low inhibition constant suggest a good inhibitory activity of the ligand. The docking score (DS, kJ mol^−1^) and the inhibition constant (K_i_, μM) of the 21 aaptamines (set C) are presented in table 1, along with the values of therapeutical references, such as donepezil (DON), sinefungin, tolcapone (TOL) and an alanine derivative safinamide (SAG). The DS values for these molecules show a broad range, i.e. from −47.70 to −27.82 kJ mol^−1^ for AChE, −41.51 to −22.38 kJ mol^−1^ for COMT and −43.56 to −23.68 kJ mol^−1^ for MAOB. Overall, aaptamines demonstrate stronger binding affinity (lower docking score) with AChE and MAOB compared to COMT. Thus, the interaction strength of aaptamines with the targets generally follows the order MAOB ~ AChE > COMT.

**Table 1.** Docking score (DS, kJ mol^−1^) and inhibition constant (K_i_, μM) between 21 aaptamines of set C and three studied enzymes (AChE, COMT and MAOB).

target	AChE		COMT		MAOB	
	DS	K_i_	DS	K_i_	DS	K_i_
**A1**	−30.12	5.31	−27.66	14.30	−31.00	3.72
**A2**	−28.49	10.16	−28.74	9.26	−32.34	2.15
**A3**	−27.82	13.40	−30.96	3.78	−30.79	4.06
**A4**	−28.24	11.25	−26.65	21.24	−33.18	1.53
**A5**	−31.84	2.65	−22.38	120.08	−32.84	1.78
**A6**	−31.17	3.48	−24.69	47.49	−33.93	1.14
**A8**	−30.29	4.91	−28.37	10.70	−31.30	3.31
**A9**	−30.29	4.92	−31.51	2.91	−32.01	2.48
**A10**	−29.87	5.38	−30.88	3.91	−31.63	2.88
**A11**	−35.86	0.525	−31.38	3.18	−37.78	0.238
**A12**	−41.05	0.064	−36.69	0.375	−43.56	0.023
**A14**	−35.27	0.659	−27.87	13.04	−39.50	0.120
**A15**	−35.82	0.534	−28.20	11.42	−39.79	0.106
**A17**	−30.50	4.53	−25.86	29.36	−32.47	2.07
**A18**	−31.97	2.51	−27.78	13.60	−34.56	0.884
**A19**	−35.23	0.671	−26.99	18.60	−38.62	0.172
**A21**	−33.26	1.50	−26.19	25.99	−35.61	0.580
**A22**	−37.03	0.324	−26.94	18.88	−37.82	0.234
**A24**	−45.23	0.011	−41.51	0.053	−26.82	20.09
**A25**	−38.20	0.201	−30.63	4.34	−23.68	71.32
**A27**	−47.70	0.008	−32.55	1.98	−30.71	4.15
DON^a^	−46.28	0.008				
SIN^b^			−51.67	0.891		
TOL^c^			−35.06	0.714		
SAG^d^					−35.98	0.499

^a^ Donepezil. ^b^Sinefungin. ^c^Tolcapone. ^d^(*S*)-(+)-2-[4-(fluorobenzyloxybenzylamino)propionamide].

For AChE, the most stable complex structure (lowest docking score) is observed with compound **A27** (−47.70 kJ mol^−1^), followed by **A24** (−45.23 kJ mol^−1^) and **A12** (−41.05 kJ mol^−1^). Notably, redocking of donepezil into the active pocket of AChE yields a stabilization energy of −46.28 kJ mol^−1^ and the complex structure closely overlaps with the one in the crystallized protein (RMSD = 0.64 Å). Not surprisingly, compounds **A27**, **A24** and **A12** also represent the lowest inhibition constants, the values being at 0.008, 0.011 and 0.064 μM, respectively. For MAOB, compound **A12** demonstrates both the lowest binding energies (−43.56 kJ mol^−1^) and the smallest inhibition constant (0.023 μM). Interestingly, its DS is also lower than the binding energy of the reference SAG. Last, for COMT, only **A24** shows low binding energies (−41.51 kJ mol^−1^) and inhibition constant (53 μM). Although the DS value of **A24** remains lower than that of the reference compound tolcapone, it exceeds that of sinefungin. In addition, based on the DS results, the two ligands **A12** and **A24** are multi-target inhibitors, simultaneously with AChE and MAOB (**A12**), or with AChE and COMT (**A24**).

More insights into key interactions like hydrogen bonds, hydrophobic contacts and electrostatic interactions, such as C–H bonding, π–π or π–σ interactions for the top inhibitors are given in electronic supplementary material, table S3. Although the different aspects of the molecular structure, some key interactions can be argued. For AChE, the complex structures are mainly stabilized by hydrogen bonding between either the =N site within the ring or the C=O group of the ligands and the amino acid Phe295. Other acceptor sites, such as H from –NH and –OH groups, can also form hydrogen bonds with residues Tyr124 (**A27**), Tyr341 (**A12**), and Ser203 and Gly122 (**A24**). The π–π stacking interactions are also observed between the rings of the aaptamines’ scaffold and the residue Trp286, or Tyr337, Tyr341. Additional interactions including C–H bonding with Arg296 (**A12**) and π–σ interaction with Ser293 and Val294 (**A27**) are also observed. For the most stable complex structure of **A24** with COMT, the phenol substituent forms a hydrogen bond between the hydroxyl group and the oxygen of residue Glu133, and a π–π stacking interaction with Trp186. While for the MAOB target, π–π conjugation interaction is noted between the aaptamine core rings and residues Tyr398 and Tyr435. Both conventional hydrogen bonds and C–H bonds form primarily with Gly58 and Tyr60 through the methoxy group.

Overall, three best candidate aaptamines for three targets are chosen, i.e. **A12**, **A24** and **A27** for MAOB, COMT and AChE, respectively. For better visualization of the structures of the selected aaptamine molecules, their optimized geometry and electronic configuration, including the highest occupied molecular orbitals, lowest unoccupied molecular orbitals and electrostatic potential (ESP) maps, are given in electronic supplementary material, figure S1.

### 3.3. Time-resolved dynamics interactions

**Figure 3.**
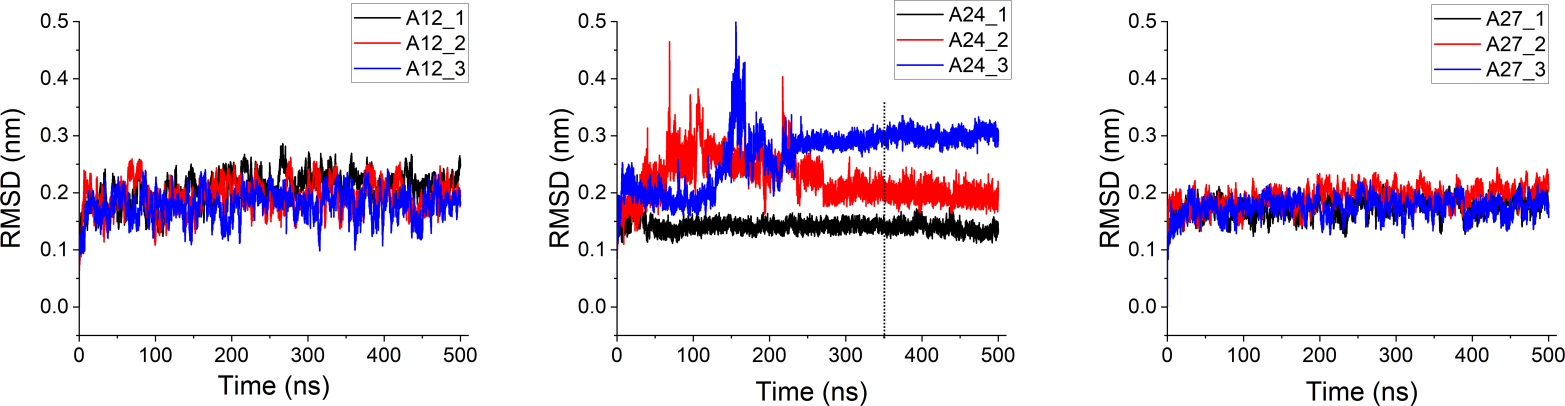
Time-dependent RMSD (nm) of the complexes in the time range 500 ns. Each complex is presented with three independent trajectories.

MD studies that can provide further insights into the interactions, conformational changes and stability of the protein–ligand complex in a more dynamical way are then performed for the three most potent complexes, i.e. **A12**@MAOB, **A24**@COMT, **A27@**AChE, as well as for their references. Thus, the ligand–enzyme interacting activity is investigated in water over a real time scale of 500 ns. Conformational changes of the systems via RMSD analysis of the heavy atoms of ligands and Cα atoms of protein, investigated with three independent trajectories for each complex, are presented in figure 3. The same analysis for the reference complexes is presented in electronic supplementary material, figure S2. The result indicates relatively good equilibration and stability of **A12** and **A27** in their complexes over the 500 ns time scale, with consistent RMSD values found around 0.15−0.20 nm. The lowest mean and less deviation value of RMSD are found for **A27** (0.181 ± 0.012 nm) and **A12** (0.195 ± 0.017 nm). Very similar data are obtained for their references, i.e. 0.205 ± 0.019 nm and 0.193 ± 0.013 nm for SAG and DON, respectively. In contrast, higher fluctuation of RMSD is observed for both **A24** (0.210 ± 0.018 nm) and TOL (0.170 ± 0.026 nm) in interacting with the COMT target. Particularly, in the second and third trajectories, a high fluctuation in RMSD values was observed (up to 0.5 nm), indicating a significant ligand mobility relative to the protein backbone. This result may be attributed to (i) the small size of COMT target compared to AChE and MAOB and (ii) the location of the catalytic active sites. Regarding the latter, while the active pockets of MAOB and AChE are located within the protein, constraining the movement of the ligands **A27** and **A12**, the ligand **A24** interacts more freely with COMT target. Since COMT’s active sites are positioned on the peripheral surface of the protein (electronic supplementary material, figure S5), it provides greater spatial flexibility for ligand movement. Based on the RMSD results, we chose snapshots in the most stable regions, i.e. from 350 to 500 ns time window for MM-PBSA analysis.

Furthermore, the RMSF and the radius of gyration are also investigated (electronic supplementary material, figure S3). In general, the RMSF results show that all systems are stable, particularly for rigid regions like alpha helices or beta sheets in proteins. The most dynamic regions of each system, corresponding to higher RMSF values, are only observed at the terminal loops in each protein. Naturally, these regions are more flexible than the other parts of the proteins. The radius of gyration (R_g_) is another useful parameter to evaluate the compactness of the molecular structure. A lower R_g_ value obtained for the **A24** complex (1.65 nm) than for **A12** and **A27** complexes (around 2.3 nm) typically indicates a more compact conformation in cases of COMT complexes. The results are in line with the size of the enzymes.

**Figure 4.**
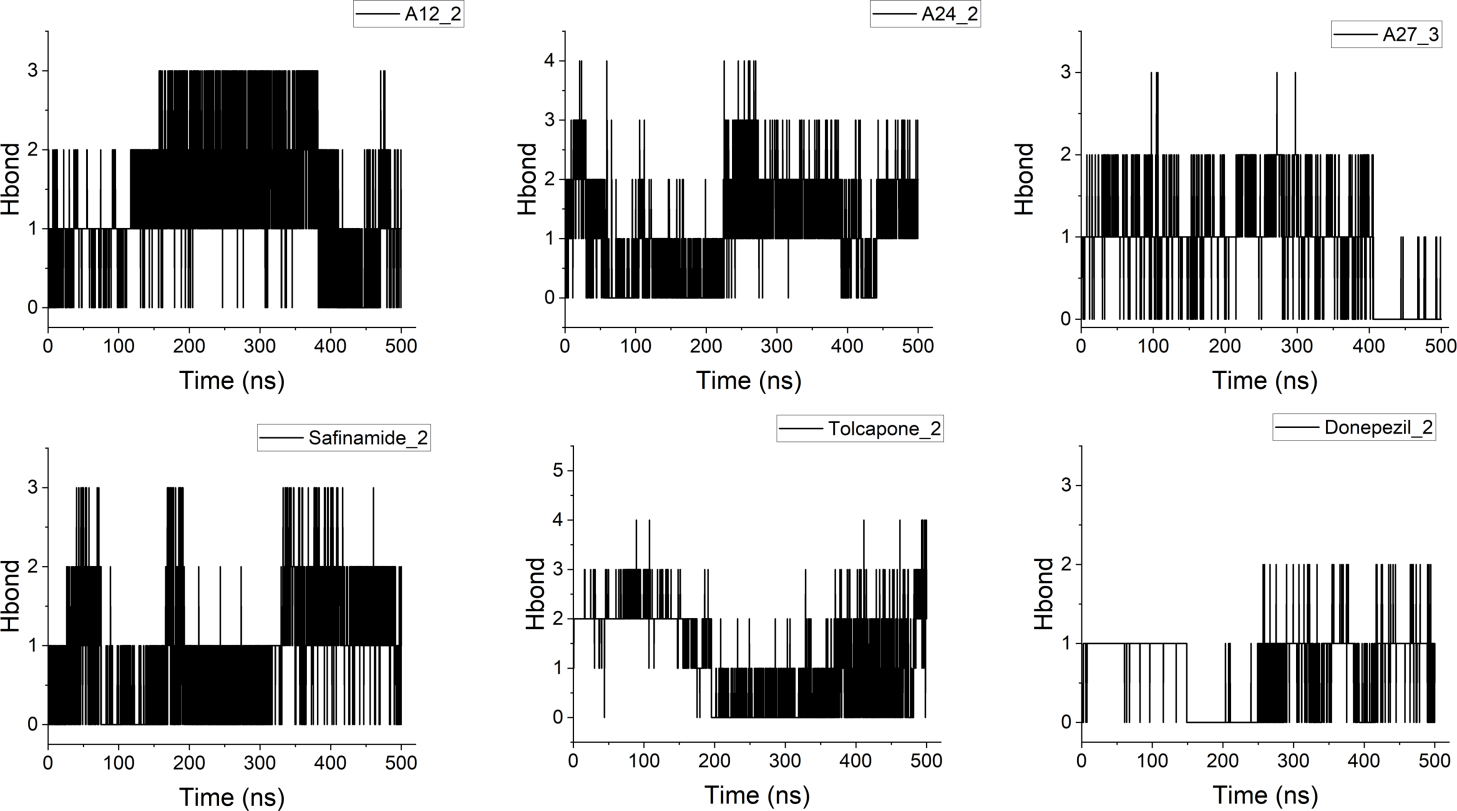
Number of H-bonds for selected trajectories of the complexes **A12**@MAOB, **A24**@COMT and **A27**@AChE (top) and their corresponding references (bottom) along the time scale of 500 ns

In general, both strong hydrogen-bond interactions and side-chain contacts can significantly contribute to complex stability. Figure 4 represents the time evolution of the number of H-bonds for the studied complexes and their references for typical trajectories, while table 2 additionally summarizes the key interactions for all studied systems. In the interaction with MAOB targets, about three hydrogen bonds are formed between the ligand, i.e. **A12** or SAG. For example, we found stable hydrogen bonds between the oxygen or nitrogen atoms of **A12** and the hydrogen of residues Tyr188, Gln206 and Tyr435. Besides, the side-chain interactions primarily involve residues Tyr326, Leu345, and Tyr398, Tyr60, Thr201, Thr174 and Pro104. In the COMT complexes, higher number of H-bonds (up to four) are observed for both **A24** and TOL. However, due to higher number of conformational changes, we can only identify some of the primary H-bonds formed with residues Lys187 and Asp188, or Glu242. The main side-chain contacts occur with different residues in the loops such as Trp81, Ala82, Met83, Trp186 and Lys187. Indeed, the loss of dynamic stability in COMT complexes results from the disruption of H-bonds between **A24** and the amino acids Lys187, Asp188 and Arg189. As the ligand shifts and obtains a straight conformation, it establishes the new H-bonds with Ser162 and Gln163. For the AChE target, fewer H-bonds formed over time, suggesting that the complex stabilization might be governed by the side-chain contacts. There are generally two H-bonds formed in the complexation, which involve N/O atoms of **A27** and H atoms of residues Tyr124, Gln291, Phe295 and Trp286. Furthermore, the primary side-chain contacts found with amino acids Tyr72, Ser125, Ser293, Val294, Tyr341 and Gly448 play an important role in stabilizing the complex structure.

**Table 2.** Main H-bonds and side-chain contacts in the studied complexes.

complexes	H-bonds	side-chain contacts
**A12**@MAOB	Tyr188, Gln206, Tyr435	Tyr60, Pro104, Thr174, Thr201, Tyr326, Leu345, Tyr398
SAG@MAOB	Tyr60, Leu171, Gln206	Phe103, Pro104, Phe168, Leu171, Thr201, Tyr398
**A24**@COMT	Lys187	Ala82, Met83, Ala110, Trp186, Lys187, Asn213, Gly218, Glu242
TOL@COMT	Asp188, Glu242	Trp81, Met83, Lys187, Val216, Pro217
**A27**@AChE	Tyr124, Gln291, Phe295	Tyr72, Ser125, Ser293, Val294, Tyr341, Gly448
DON@AChE	Tyr124, Trp286, Tyr337	Tyr72, Tyr86, Tyr124, Trp286, Ser293, Phe338, Tyr341, Gly342

In terms of energy, the MM-PBSA method is used to estimate the binding free energy of the complexes (table 3). Direct interacting components such as electrostatic and van der Waals energies, polar solvation energy and non-polar solvation energy by accessible surface area energies are interpreted. First and foremost, it can be seen that the studied aaptamines similarly (**A12**) or more strongly (**A24** and **A27**) bind to their target. In particular, **A27** shows the lowest binding energy Δ*G*_bind_ to AChE (−170.42 ± 14.24 kJ mol^−1^). This result suggests that aaptamine **A27** might be the best candidate for ND treatment. The lowest Δ*G*_bind_ obtained in **A27** results from a low polar solvation energy (34.52 ± 7.87 kJ mol^−1^) compared to other ligands, while the highest Δ*G*_PB_ obtained in the case of donepezil (170.55 ± 13.01 kJ mol^−1^) also raises the Δ*G*_bind_ of its complex to −77.13 ± 10.04 kJ mol^−1^. Second, it is clear that the binding activity of all the ligands to their target is mostly driven by the van der Waals energy. Indeed, Δ*E*_vdW_ are found from −87.20 ± 3.16 (TOL) to −193.04 ± 6.69 kJ mol^−1^ (DON), while the electrostatic energies Δ*E*_elec_ are only from −22.64 ± 9.72 (**A12**) to −74.29 ± 17.66 kJ mol^−1^ (SAG). In each complex, the Δ*E*_vdW_ is from 36 to 160 kJ mol^−1^ lower than the Δ*E*_elec_, making the van der Waals interaction the key component in the ligand–enzyme binding. In our study, the entropy of the complex systems is lowest in the case of interaction with MAOB, estimated as 23.05 ± 5.4 and 25.44 ± 5.10 kJ mol^−1^, for **A12** and SAG, respectively. As expected, the highest entropy is found for COMT target, with values at 45.22 ± 1.19 and 40.84 ± 8.20 kJ mol^−1^, for TOL and **A24**, respectively. This result is totally in agreement with the above RMSD and RMSF analysis of the complexes.

**Table 3.** Electrostatic (Δ*E*_elec_) and van der Waals (Δ*E*_vdW_) energies, polar solvation energy (Δ*G*_PB_), non-polar solvation energy (Δ*G*_SA_), entropy (−*T*Δ*S*) and binding energy (Δ*G*_bind_) of the studied complexes estimated from he MM-PBSA method. All energies are in kJ mol^−1^.

complexes	Δ*E*_elec_	Δ*E*_vdW_	Δ*G*_PB_	Δ*G*_SA_	−*T*Δ*S*	Δ*G*_bind_
**A12**@MAOB	−22.64 ± 9.72	−182.38 ± 1.01	120.88 ± 14.70	−23.47 ± 0.62	23.05 ± 5.4	−84.54 ± 16.69
SAG@MAOB	−74.29 ± 17.66	−188.70 ± 3.64	166.69 ± 8.20	−25.20 ± 0.25	25.44 ± 5.10	−96.02 ± 5.44
**A24**@COMT	−73.99 ± 19.10	−132.98 ± 26.96	134.58 ± 37.90	−19.81 ± 2.58	40.84 ± 8.20	−51.36 ± 20.27
TOL@COMT	−50.53 ± 25.83	−87.20 ± 3.16	71.86 ± 22.91	−13.78 ± 0.86	45.22 ± 1.19	−34.41 ± 5.75
**A27**@AChE	−31.15 ± 7.36	−189.37 ± 7.80	34.52 ± 7.87	−24.67 ± 2.24	40.26 ± 5.86	−170.42 ± 14.24
DON@AChE	−54.37 ± 5.40	−193.04 ± 6.69	170.55 ± 13.01	−29.28 ± 0.13	29.02 ± 7.57	−77.13 ± 10.04

**Figure 5.**
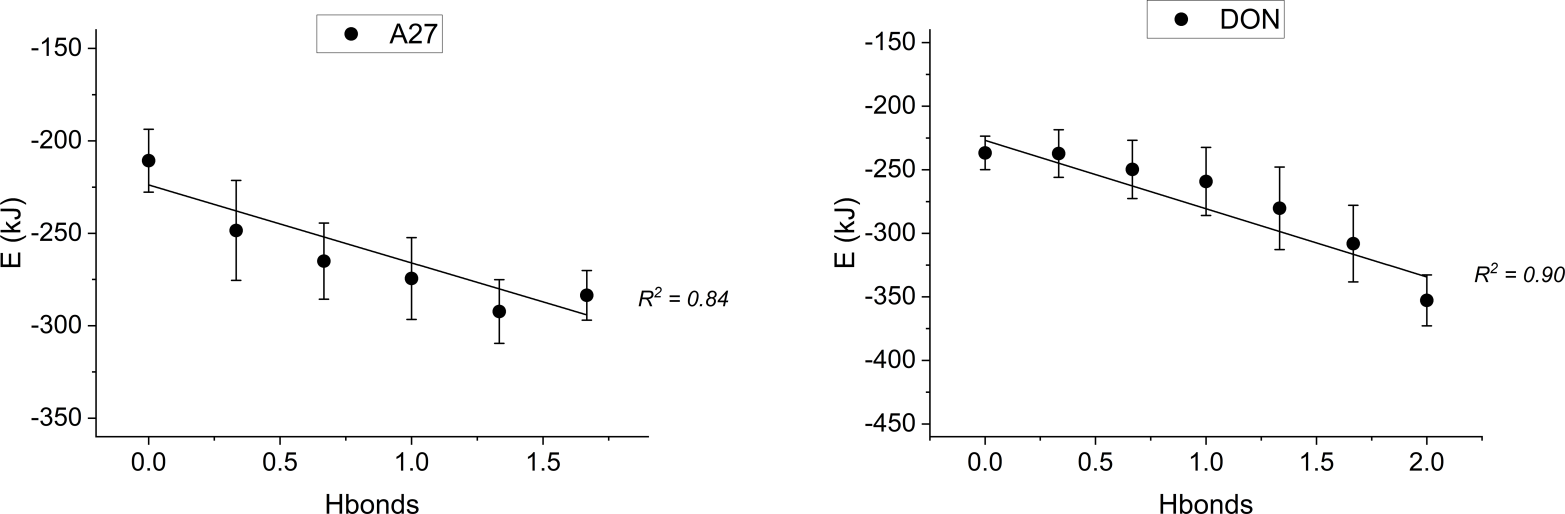
Total energy of **A27**@AChE and DON@AChE in relationship with the number of H-bonds of the complexes.

Particularly, the formation of H-bonds between aaptamine ligands and studied targets also plays an important in stabilizing the total energy of the complexes. Indeed, increasing the average number of H-bonds between **A27** and DON with AChE helps to decrease the total energy of the complexes. This relationship is supported by a strong linear regression, with *R*^2^ coefficient values being 0.84 and 0.90, for **A27** and DON, respectively (figure 5). Similar findings for MAOB and COMT complexes are given in electronic supplementary material, figure S4.

**Figure 6.**
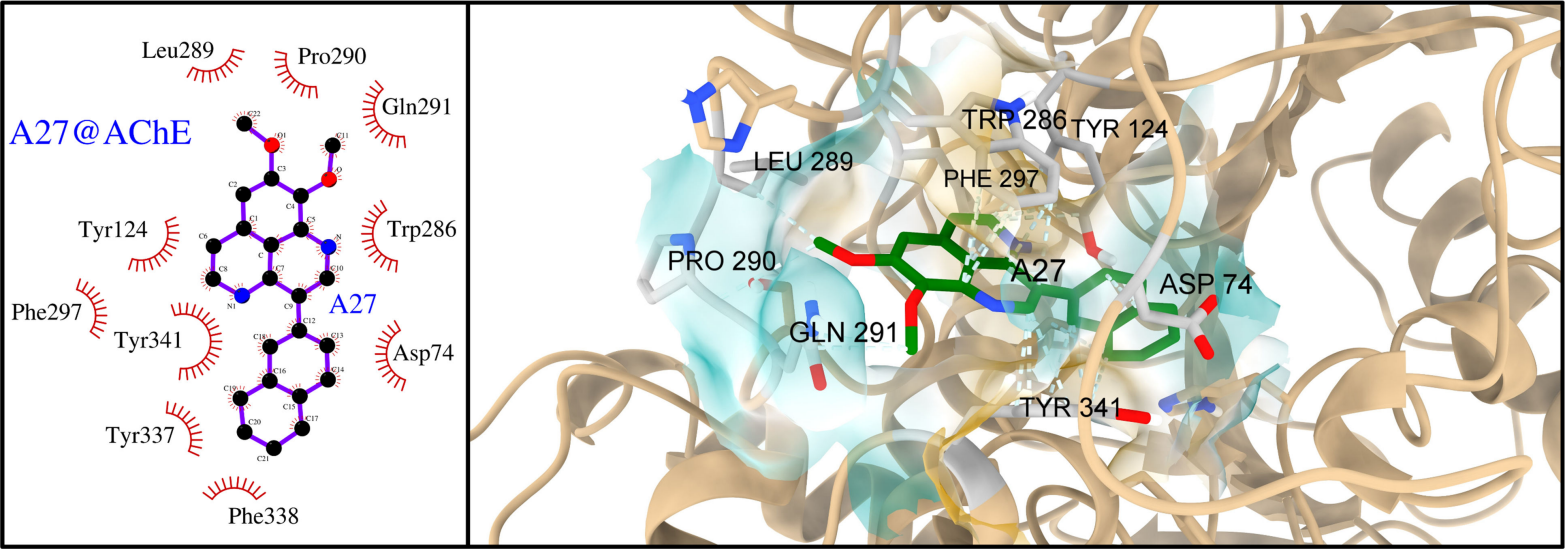
2D and 3D illustrative images of the binding pose of **A27** in AChE. The results are obtained by clustering the Cα and heavy atoms of ligand with a cut-off 0.25 nm using MD snapshots.

The clustering analysis was performed using gmx cluster with the GROMOS method, which groups similar conformations of the complexes. This approach identifies the most characteristic conformations of the ligand–enzyme complexes. The 2D and 3D images display the binding poses of the best inhibitor, **A27**, and its receptor, obtained from the centroid structure of the largest cluster as illustrated in figure 6. One can see that ligand **A27** remains well positioned within the AChE active pocket, building by the amino acids Asp74, Tyr124, Phe297, Leu289, Pro290 and Gln291 of the loops, and part of the helices Tyr337, Phe338 and Tyr341. Our results are consistent with previous reports on quinoline derivatives, for which DON, as well as quinoline inhibitors, occupy a similar cavity form by both catalytic active site (CAS) and peripheral anionic sites (PAS) of AChE [72]. Similarly, 2D and 3D images showing interactions of other ligands in their cluster structures are presented in electronic supplementary material, figures S4–S6, for MAOB, COMT and AChE targets, respectively. Overall, these MD simulations are in agreement with the initial docking results, demonstrating that the aaptamines fit the catalytic site of the enzymes, showing similar or better binding activity than the references, and the complexes remain stable over 500 ns in water.

**Figure 7.**
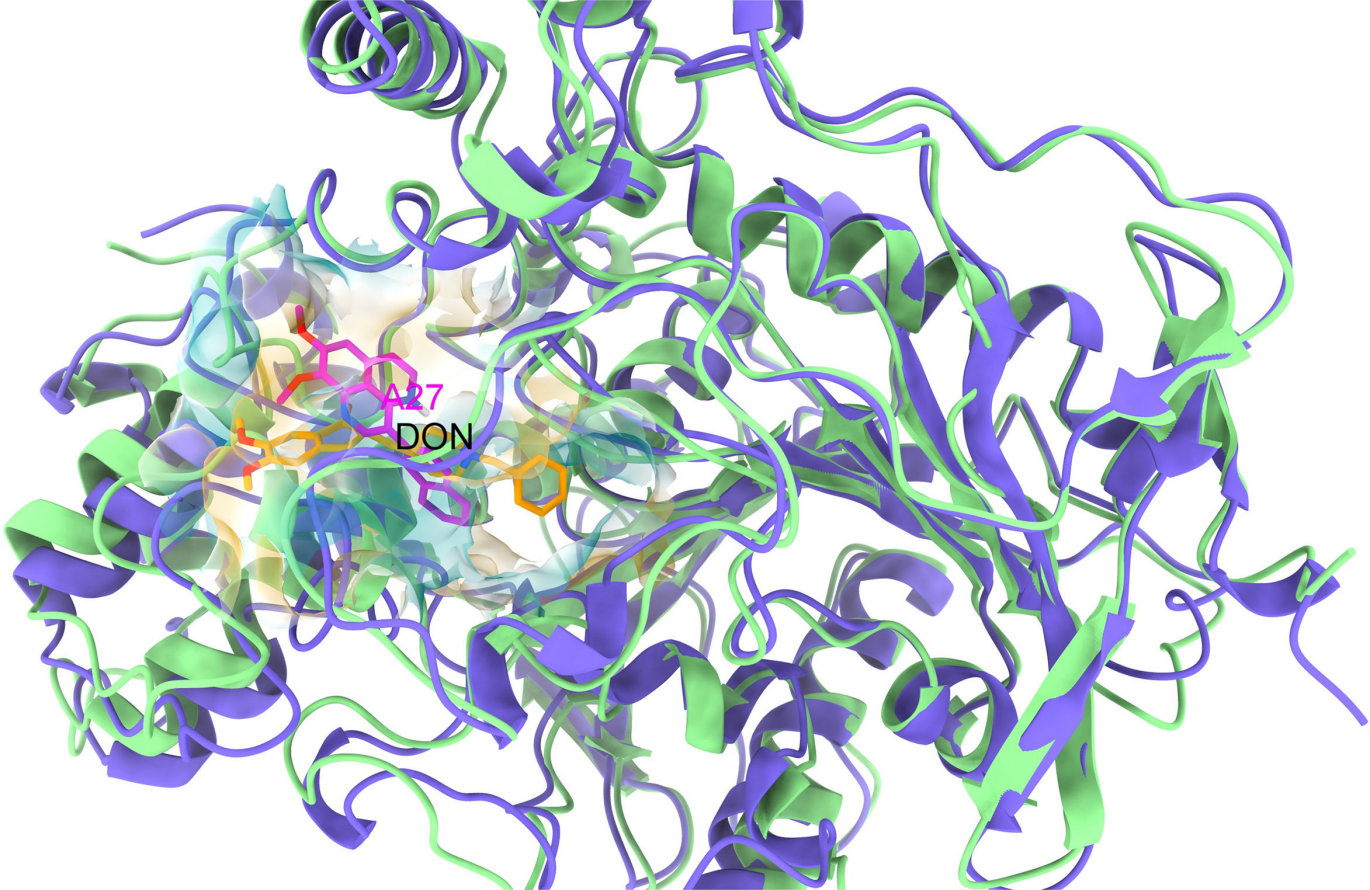
The occupied cavities of **A27** (magenta) and DON (gold) in the catalytic active sites of AChE after alignment.

Finally, comparing the **A27** conformation with the one of DON in the active pocket of AChE demonstrates that both ligands, with similar size and planarity, fit into almost the same cavity (figure 7). Structural analysis shows that two ligands share several similar features, for example, the presence of two methoxy groups attached to an aromatic ring at one terminal, and an electron-withdrawing group at the other end. Notably, for AChE, three aaptamines (**A12**, **A24** and **A27**) show the lowest docking scores. A similarity search employing the Tanimoto method and RDKit topological fingerprint reveals that the structure of **A12** and **A24** shows a similarity of 76% and 78%, respectively, to that of **A27**. The important structural features might include a ketone or methoxy groups (C=O/OCH_3_) at C8 or C9 position, and an additional electron-withdrawing group at C3 position. Therefore, we suggest that the molecular design of new AD drugs based on the aaptamine scaffold should consider modifying the functional groups at C3, C8 and C9 positions.

## 4. Conclusions

In conclusion, we performed a three-tier study to identify potential aaptamines from a library of 28 compounds that exhibit enzyme inhibition activities for the treatment of NDs. Three enzymes generally associated with neurodegenerative conditions, AChE, MAOB and COMT enzymes, were tested. The hit compounds were first selected based on the physicochemical, drug-like and pharmacokinetics properties and static binding energy. The three lowest docking score complexes were identified as **A12**@MAOB, **A24**@COMT and **A27**@AChE. Finally, MD studies the conformational changes, the stability, and the detailed interactions of these complex systems on a real time scale (500 ns). The results show that all studied complexes are stable in water during that period. Moreover, they are also comparable to (**A12**) or better than (**A24**, **A27**) the therapeutic references in terms of binding energy to the corresponding targets, with the predominant contribution via van der Waals interactions. Our study not only identifes **A27** as the best candidate for further study of ND treatment but also reveals key structural features for molecular drug design using the unusual aaptaminoid scaffold. Based on the similarity of **A12**, **A24** and **A27** structures, we suggest that modification of the aaptaminoid structures should focus on introducing electron-withdrawing groups at C3 position, while keeping the methoxy and/or carbonyl substitution at C8 and C9 positions. This strategy can be of great value in CADD as well as in pharmaceutical synthetic processes.

## Data Availability

All relevant data to reproduce all results in the paper are in the main text. Additional data are available on Dryad [73]. Electronic supplementary material is available online [74].
